# Anthropometric and Physical Performance Profiles of Elite Karate Kumite and Kata Competitors

**DOI:** 10.2478/v10078-011-0078-x

**Published:** 2011-12-25

**Authors:** Nenad Koropanovski, Bobana Berjan, Predrag R. Bozic, Nemanja Pazin, Aleksandra Sanader, Srecko Jovanovic, Slobodan Jaric

**Affiliations:** 1 The Research Center, Faculty of Sports and Physical Education, Belgrade University, Serbia; 2 Serbian Institute of Sport, Belgrade; 3 Department of Special Physical Education, Academy of Criminalities and Police Studies, Belgrade, Serbia; 4 Department of Kinesiology and Applied Physiology, University of Delaware, Newark, USA

**Keywords:** testing, training, selection, morphology, karate, motor abilities

## Abstract

Karate tournaments consist of two equally important karate disciplines: the kumite and kata competitions. Due to being based both on the distinctive selection of movement techniques and their kinematic and kinetic patterns, we hypothesized that the elite kumite and kata competitors could differ regarding their anthropometric and physical performance profiles. Thirty-one senior male karate competitors of the national karate team (kumite n = 19; kata n = 12) participated in this study. The tests applied included both the assessment of anthropometric (body height, mass and body mass index) and the following physical performance measurements: the adductor and hamstring flexibility (sideward leg splits test), speed and acceleration (20-m sprint test with 10-m acceleration time), explosive power (countermovement and standing triple jump), agility (“T”- test) and aerobic endurance (20-m multistage shuttle run test). The kumite competitors revealed a larger body size through body height (p = 0.01) and mass (p = 0.03), while the differences in body composition were non-significant. The kumite competitors also demonstrated higher acceleration (p = 0.03) and explosive power (standing triple jump; p = 0.03). A 6-7° higher flexibility of the kata competitors remained somewhat below the level of significance (p = 0.09). The findings could be interpreted by the distinctive differences in the movement techniques. Specifically, a higher explosive power could be beneficial for kumite, while both a smaller stature and higher flexibility (particularly of the lower extremity) could be important for the exceptionally low postures of the kata competitors. Although further elucidation is apparently needed, the obtained finding could be of importance for both the early selection and training of karate competitors.

## Introduction

Karate is arguably one of the most popular martial arts practiced worldwide. Traditional karate training involves basic techniques, kata, and sparring activities (Imamura, 1998). Modern non-contact karate tournaments of the World Karate Federation consist of two equally important karate disciplines: the kumite and kata. Kumite is a synonym for karate fight and consists of the execution of freely chosen defensive and offensive techniques applied against an opponent (Imamura, 2002). In general, the kumite consists of burst of consecutive techniques separated by intermittent hopping movements that allow for rapid changes of body position (Iide, 2008). The kata performance consists of prescribed sequences of defensive and offensive techniques. While following the prescribed movement sequences, the techniques are rather formal, systematic, predominantly slow and mainly performed in relatively low postures, (Imamura, 1998).

In past, the demands of karate competitions used to be similar for both disciplines, as well as for the standard training programs. As a consequence, the participants often used to successfully compete in both the kata and kumite. However, the alternation of the competition rules (e.g. by restricting contacts, transition from one point to a multi-scoring system, or allowing a higher scoring of leg techniques) has made the kumite competitions both more dynamic and attractive (Macan, 2006). Unlike the kumite, the kata competition has not been evolved much since the basic requirements remained virtually unchanged. As a consequence, a specialization of elite karate competitors for kata and kumite has become more prominent. Presently, the kumite is almost completely separated from the kata techniques and, consequently, only occasionally some young participants still compete in both disciplines.

In general, the selection of athletes should be primarily based on the abilities and skills that have a crucial influence on sport performance, where genetic factors could be of considerable importance (Vaeyens, 2008). Taking into account the evident current differences in demands between the kumite and kata techniques, as well as a widespread popularity of karate, it seems surprising that the differences in anthropometric characteristics and physical abilities between the kumite and kata competitors have been rarely studied. Dealing with the somatotype, Fritzschel (2007) explored the anthropometric differences between elite kumite and kata athletes. They found that the kumite and kata competitors could be relatively more endomorph and ectomorph, respectively. Ravier (2004; 2006) investigated the relationship between various test movement performance and the blood markers of anaerobic metabolism in two different categories of kumite competitors in order to propose a valid kumite-specific test battery. They found that power, speed, as well as the ammonia and lactate accumulation, could be sensitive enough to detect the difference in performance level. Finally, Blazevic (2006) attempted to identify the ‘motor structures’ that are relevant for competition success in kumite and found that speed and power were the most important abilities.

The literature reviewed above suggests that despite the general importance of physical abilities and anthropometric characteristic for the purpose of both, sport selection and evaluation of the training process (MacDougall, 1991; Sterkowicz, 2009), there is an apparent lack of data regarding the differences between kumite and kata competitors. Therefore, within the present study we evaluated the basic anthropometric dimensions and physical performance of these two groups of elite male karate competitors. Although the lack of previous research does not allow for formulating a specific hypothesis, we expected that possible differences could reflect the essential differences in competition techniques between kumite and kata. Specifically, one could expect that due to apparently needed rapid performance, the kumite competitors could reveal a higher movement speed and greater power. Conversely, due to the disadvantage of a large body size when maintaining difficult and strength demanding postures (Lohman, 1998; Jaric et al 2005) such as those required in a number of kata techniques, one could expect a smaller body size and a higher flexibility of the lower extremity in kata competitors. The expected findings could be of importance not only for early selection and training in karate, but also for designing discipline specific testing batteries for evaluation of kumite and kata competitors.

## Methods

### Subjects

Thirty-one elite senior male members of the national karate team participated in this study. Nineteen of them were medalists at the junior (16–18 years of age) and senior World, European and Balkan championships. Nineteen of them were kumite, while 12 were kata competitors (Table 1). None of them reported any medical problem or recent injuries that could compromise the tested performance. The study was approved by the Ethical Committee of the National Institute of Sport. Prior to the experiment all participants received a complete explanation regarding the purpose and procedures of the study, as well as the possible risks. They signed an informed consent document according to the Helsinki Declaration.

### Procedure

The present study is an experimental one based on a cross-sectional approach. We particularly focused on those characteristics that could differ between two groups regarding the requirements of their respective discipline-specific movements (Pauole, 2000; Ravier, 2006; Fritzschel, 2007).

Subjects were instructed to avoid any strenuous physical activity two days prior to the experiment to minimize the possible effect of fatigue. The experiment was carried out within a single testing session. It included anthropometric measurements and medical screening, followed by physical performance testing.

Body height (BH) and body mass (BM) were measured to the nearest 0.5 cm and 100 g, respectively. Thereafter, the body mass index was assessed (BMI = BM/BH^2^). Anthropometric measurements were taken by the same experimenter according to standard procedures.

The testing of physical performance was preceded by a standard 10-min warm-up and 10-min active stretching. Following a detailed explanation and qualified demonstration of each test, all subjects performed one practice trial followed by two consecutive experimental trials and the better result was used for further analysis. The rest periods between consecutive trials and between two consecutive tests were 2 and 5 min, respectively.

The evaluated physical performance included the tests of flexibility, speed, agility, power and endurance. The applied tests are described below in details.

#### Flexibility

*Sideward Leg Splits Test* (SdLS) was selected for direct assessment of the flexibility of lower extremity. It predominantly assesses the flexibility of the hamstring muscles of the front leg and the adductor muscles of the back leg (Bozic, 2010). The SdLS was selected because a number of karate techniques are performed with the hip joints exploiting the full range of motion. Specifically, the participant stands on a smooth board and supports himself with both hands. His back foot is turned out and forms an angle of 90° with the forward foot. Thereafter, he slowly slides both feet apart. The trunk remains upright (i.e. aligned with a vertical line drawn at the wall) and no hip rotation is allowed. The examiner measures the height of the symphysis with respect to the ground (h), and the distances from the vertical projection of symphysis to the heel of the back (a) and to the heel of front leg (b). Standard kinanthropometry and ruler were used for measuring the distances. Precision of the measurement was 0.5 cm. The angle formed by the legs is assessed by means of a trigonometric formula [α = atan(a/h) + atan(b/h)] and reported in degrees. Both the right and left leg forward were measured. We recently found high intra-trial and test-retest reliability (ICC > 0.9), as well as a high concurrent and factorial validity of this test (Bozic, 2010).

#### Acceleration and speed

*A 20-m sprint test* was used with separately timed first and second 10 m intervals for the assessment of acceleration (first 10 m sprint – 10S) and maximum speed over a short distance (10 m flying start – 10FS). Although the movement is not karate-specific, the test selection was based on the presumed importance of rapid movement initiation and speed for elite kumite competitors (Blazevic, 2006). The subjects were instructed to run from the standing posture as fast as possible until passing the 20 m mark. Electronic timing gates were used to evaluate these variables (Fitro Light Gates, Fitronic, Bratislava, Slovakia). Precision of the measurement was 0.001 s. Mirkov (2008) found high intra-trial reliability of a similar version of this test performed over 30 m.

#### Agility

*The T-test* (TT) was selected for the assessment of agility. It has been recently reported that the ability to change direction could be of high importance for success in martial arts (Mirkov, 2008; Blazevic, 2006). The TT course consists of two 10 m straight sections forming the shape of letter T. It includes a forward sprint (10 m), side shuffle to the left (5 m), side shuffle to the right (10 m), side shuffle back to the left (5 m), and back peddled 10 m back to the start. The same equipment as in the previous test was used for measuring the test time. Pauole (2000) reported a high intra-trial reliability of the test (ICC = 0.98).

#### Power

*Countermovement jump* (CMJ) and *Standing triple jump* (STJ) were used for indirect assessment of explosive power of leg extensors in the vertical and horizontal plane. The tests were selected because of their presumed validity for the assessment of performance of lower limbs in karate competitors (Voight, 1990; Zehr, 1997; Lee, 1999). It could also be of importance that the explosive action of the hip and knee extensor is essential for both the karate ‘stepping’ and leg kicks. Finally, note that both tests are expected to provide body size-independent indices of muscle power output and, therefore, the normalization of the recorded data is not required (Markovic, 2007; Nedeljkovic, 2009).

CMJ – The subjects were instructed to jump as high as possible by performing a preceding countermovement with arms swing. Subjects were also required to land approximately at the point of the take-off. The test was conducted on a contact platform (Contact plate, Globus, Codogne, Italy; accuracy ± 0.001 second) that records flight time (t). The rise of the center of gravity above the ground (height in meters) was measured from time of flight (t; in s) applying the ballistic law: h = ⅛ t^2^g (g = 9.81 m/s^2^). A high intra-trial and test-retest reliability of CMJ have been reported (ICC>0.9) (Markovic, 2004; Slinde, 2008).

STJ - The subjects were instructed to jump as far as possible performing a standing triple jump from a standard standing position. The distance from the starting point to the landing point at the heel contact was used for further analysis. The precision of the measurement was 1 cm. Markovic (2004) found a high intra-trial reliability (ICC = 0.93) and factorial validity of this test (r = 0.80).

#### Aerobic endurance

*20-m Shuttle run test* (SR) was used for the assesment of aerobic endurance. With the exception of the scoring system, the original test protocol of the multistage Shuttle run test was followed (Léger, 1988). In short, the subjects were instructed to run back and forth between cones set 20 m apart. The running pace was determined by audio signals emitted from a pre-recorded tape cassette. The test was set for the initial velocity of 8.0 km/h, increasing by 0.5 km/h every minute. Subjects were instructed to complete as many runs as posible. The test ended when the subject was not able to reach the cone in time in 2 consecutive runs, or when the subject felt unable to continue. Unlike the orginal test procotol which used a scoring system of “paliers” (each “palier” lasting approximately 1 min) (Léger, 1982; Léger, 1988), we scored the test by adding up the distance. Léger and Lambert (1982) found a high test-retest reliability of SR (r = 0.98). In addition, the same authors showed a high correlation between the SR score and directly assessed maximum oxygen uptake (VO_2_). Note that Beneke and co-workers (2004) recently stressed the importance of aerobic pathways for karate training.

### Statistics

Standard descriptive statistics (mean and standard deviation) were calculated for each variable. Significant statistical differences between the two groups were tested by means of the two-tailed, independent t-test. Normality of distribution of residuals was tested by means of the Kolmogorov-Smirnov test. Statistical significance was set at *p* = 0.05. All statistical tests were performed using SPSS 16.0 (SPSS INC, Chicago, IL).

## Results

Overall, the data suggest a larger body size of the kumite athletes, while the difference in body composition as assessed by BMI was not statistically significant (Table 1).

The Kolmogorov-Smirnov test revealed violation of normal distribution in none of the tested variables (Table 2). The kumite competitors revealed higher initial acceleration (10S) and higher explosive power in the test performed in the horizontal (STJ), but not vertical (CMJ) direction. Although somewhat below the level of statistical significance, the kata competitors revealed a higher flexibility. The remaining differences in individual physical performances tests were not statistically significant.

## Discussion

Within the present study we aimed to test the anthropometric and physical performance profiles of elite karate kumite and kata athletes in order to identify the differences that could be of importance for future selection, training and testing of karate competitors. The findings were mainly in line with our hypotheses based on the differences in both the selection and the patterns of typical techniques performed by kumite and kata competitors.

The recorded anthropometric variables (BH, BM and BMI) of the tested karate athletes were comparable with those of elite karate athletes tested in earlier studies (Ravier, 2004; Zemakova, 2004; Ravier, 2006; Fritzschel, 2007).

However, it should be kept in mind that both the range and distribution of body size of the kumite competitors tested in our study were inevitably affected by the distribution of weight categories prescribed by the competition rules. Nevertheless, presuming that weight categories correspond to body size distribution of the general population of kumite competitors (as basically intended), our results revealed a higher body size in kumite than in kata competitors. Part of explanation could be found in the effects of body size scale that selectively affect the different physical performance (c.f., McMahon, 1984). Specifically, while the maximum movement velocity (an ability crucial for success in kumite competition) should mainly remain unaffected by body size (Jaric, 2005), the kumite competitors should benefit from superior longitudinal body dimensions enabling earlier reaching and punching an opponent, especially in intercepting actions. However, a typical kata competition involves a number of traditional karate postures that are rather low and, therefore, quite strength demanding. Since muscle strength increases with body size at a lower rate than body weight (Jaric, 2005), the kata competitors could benefit from a smaller stature. Note that the same rationale has been frequently used to explain a relatively small body size of acrobats or gymnast (McMahon, 1984).

Regarding physical performance, we found a higher ability to accelerate the whole body (as assessed by both the 10S and STJ test) in the kumite competitors. Again, the obtained difference could be explained by the specific requirements of the typical kata and kumite competitions. Specifically, a success of both the attacking and defensive kumite techniques highly depends on the ability to rapidly initiate the change of body position in horizontal direction. The kata competitors, however, are expected to demonstrate an excellence in kinematic patterns of the prescribed techniques performed at a moderate pace what does not require rapid changes in body position.

Regarding the remaining tests of maximum performance, one could speculate that a lack of differences in CMJ between two groups could be a consequence of a need of the kumite competitors to rapidly change the body position in horizontal, but not in vertical direction. Note that only a moderate relationship (i.e. the correlation coefficients between 0.46 and 0.77) were found between the abilities to accelerate in vertical (i.e. vertical jump) and horizontal direction (i.e. sprinting) (Young, 1995; Nesser, 1996; Kukolj, 1999; Maulder, 2005), while a stronger relationship was found between the running and long jump performance (r = −0.86) (Maulder, 2005).

We did not find differences between the two groups regarding aerobic endurance (SR), agility (TT) and flexibility (SdLS). The former finding could be explained by similar aerobic demands of the kata and kumite during both training and competition or, alternatively, by a relatively low sensitivity of the applied test (Bangsbo, 1992; Imamura, 1998; Bangsbo, 2008). Therefore, we recommend using more specific endurance tests in future assessments, particularly those based on intermittent activity that would correspond to the duration of a typical karate competition (Krustrup, 2006; Bangsbo, 2008). Regarding TT test, the lack of difference between two groups could be somewhat surprising since only the kumite competition requires rapid changes in movement direction. Therefore, it remains possible that other factors, such as anticipation and pattern recognition (Sheppard, 2006), or a selection of proper movement techniques could be relatively more important for a kumite competitor than the ability to rapidly change movement direction. Finally, although somewhat below the level of statistical significance, note that the kata competitors revealed 6–7° higher flexibility of lower limbs than the kumite competitors. Once again, we believe that the differences in the techniques could explain this finding. Namely, while the kumite requires the movement techniques to be performed without strictly prescribed kinematic patterns, the kata competition is based on low postures that require high flexibility of both the hamstring and the hip abductor muscles. Since the observed difference could have reached the level of statistical significance on a larger sample of participants, we recommend SdSL for further use in evaluations of karate athletes.

To conclude, the findings suggest that kumite competitors could be of larger body size, higher ability for initiation of body movement in horizontal direction and, possibly, relatively reduced flexibility of lower limbs. Since only elite competitors were tested, the findings could be of importance for both the selection and training design of karate athletes. Future elucidation of the discipline specific profiles of the kumite and kata competitors could include larger numbers of the anthropometric measures, physical performance and skill-specific tests, as well as the extension of the same research to female competitors. However, based on the essential differences in the skills required from elite kumite and kata competitors (i.e. closed and predominantly proactive vs. open and predominantly reactive, respectively), a particularly promising extension of this line of research could be towards more neurophysiologicaly oriented tests, such as the reaction time and the ability for a rapid stimulus-reaction processing.

## Figures and Tables

**Table 1:** Demographic and anthropometric profiles of the karate competitors

	Kumite (N = 19)		Kata (N = 12)		*p-value*
	Mean	SD	Mean	SD	
Age (years)	21.0	2.8	20.7	4.4	0.80*
Body Height (cm)	181.3	8.0	174.3	5.5	0.01*
Body Mass (kg)	77.6	10.9	70.5	5.0	0.04*
Body Mass Index (kg/m^2^)	23.5	2.1	23.2	1.8	0.68*

* - significant difference between groups (independent t-test)

**Table 2:** Physical performance measures of the kumite and kate competitors (mean and SD).

	Kumite (N = 19)		Kata (N = 12)		*p*
	Mean	SD	Mean	SD	
SdLS right (º)	150.4	12.0	156.5	10.3	0.15
SdLS left (º)	148.8	10.9	155.8	10.5	0.09
10S (s)	1.80	0.05	1.86	0.07	0.03*
10FS (s)	1.30	0.03	1.31	0.06	0.35
TT (s)	10.83	0.28	10.91	0.48	0.59
CMJ (cm)	46.1	4.4	48.6	8.1	0.35
STJ (m)	7.24	0.25	6.82	0.43	0.03*
SR (m)	1938	342	1873	367	0.42

SdLS – sideward leg splits; 10S – 10 m maximum acceleration sprint; 10FS – 10 m flying sprint; TT – T-test; CMJ – countermovement jump; STJ – standing triple jump; SR – shutlle run.

* - significant difference between groups (independent t-test)
